# The Emerging Role of miR-223 in Platelet Reactivity: Implications in Antiplatelet Therapy

**DOI:** 10.1155/2015/981841

**Published:** 2015-06-28

**Authors:** Rui Shi, Xin Zhou, Wen-Jie Ji, Ying-Ying Zhang, Yong-Qiang Ma, Jian-Qi Zhang, Yu-Ming Li

**Affiliations:** Tianjin Key Laboratory of Cardiovascular Remodeling and Target Organ Injury, Institute of Cardiovascular Disease and Heart Center, Pingjin Hospital, Logistics University of the Chinese People's Armed Police Forces, 220 Cheng-Lin Road, Tianjin 300162, China

## Abstract

Platelets are anuclear cells and are devoid of genomic DNA, but they are capable of *de novo* protein synthesis from mRNA derived from their progenitor cells, megakaryocytes. There is mounting evidence that microRNA (miRNA) plays an important role in regulating gene expression in platelets. miR-223 is the most abundant miRNAs in megakaryocytes and platelets. One of the miR-223-regulated genes is ADP P2Y12, a key target for current antiplatelet drug therapy. Recent studies showed that a blunted response to P2Y12 antagonist, that is, high on-treatment platelet reactivity (HTPR), is a strong predictor of major cardiovascular events (MACEs) in coronary heart disease (CHD) patients receiving antiplatelet treatment. Recent clinical cohort study showed that the level of circulating miR-223 is inversely associated with MACE in CHD patients. In addition, our recent data demonstrated that the level of both intraplatelet and circulating miR-223 is an independent predictor for HTPR, thus providing a link between miR-223 and MACE. These lines of evidence indicate that miR-223 may serve as a potential regulatory target for HTPR, as well as a diagnostic tool for identification of HTPR in clinical settings.

## 1. Introduction

MicroRNAs were first identified in* C. elegans* in 1993 by the laboratories of Victor Ambros and Gary Ruvkin [1]. They are small noncoding RNAs that regulate gene expression from viruses to humans [2]. Approximately 30% of miRNAs genes are located in intergenic regions, and approximately 70% are located within introns or exons of protein coding genes [3, 4]. It has been estimated that miRNAs regulate between one-third and two-thirds of the human genome and are involved in most of the cellular functions [5, 6]. So far, more than 500 human miRNAs have been identified, and roughly 1000 are estimated to exist [7, 8]. Some miRNAs are expressed ubiquitously, but many are tissue and/or developmental stage specific [9]. Many studies have demonstrated that miRNAs expression is associated with disease (for different types of cancer, inflammatory disease, autoimmune disease, and cardiovascular disease) [10–14]. miR-223 has a key role mainly in the development and homeostasis of the immune system and hematological system [3]. In this review, we will discuss current knowledge of miR-223, with focus on its emerging role in the platelet function regulation.

## 2. miRNA Biogenesis and Function

miRNA genes are transcribed from the genomes of nucleated cells into primary miRNAs (pri-miRNAs), which are trimmed into miRNA precursors (pre-miRNAs) by the nuclear RNase III Drosha [15, 16], acting together with the DiGeorge syndrome critical region 8 (DGCR8) protein within the microprocessor complex [17]. The pre-miRNAs are transported out of the nucleus via exportin 5. In the cytoplasm, the 3′ overhang of the pre-miRNA is recognized by the Dicer-TAR RNA-binding protein (TRBP) complex [18]. Assisted by TAR RNA-binding protein 2 (TRBP2), Dicer cleaves the stem of pre-miRNA substrates at the base of the loop to generate miRNA-miRNA^*^ duplexes. Dicer is another RNase type III endonuclease, which generates the miRNA duplex for many miRNAs. The strands separate and the mature miRNA associates with a macromolecular complex called the RNA-induced silencing complex (RISC), which guides the miRNA to its mRNA target. The mature miRNAs are subsequently incorporated into effector ribonucleoprotein (RNP) complexes containing Argonaute 2 (Ago2) and fragile X mental retardation protein (FMRP), guiding the miRNPs for the regulation of specific mRNAs [19, 20]. The process of miRNA generation is regulated at both transcriptional and posttranscriptional levels, occasionally composed of positive feedforward or negative feedback loop, in which the miRNA targets a transcriptional activator or repressor of itself [21].

A fundamental aspect of miRNA function relates to mRNA targeting: most miRNAs are predicted to target multiple mRNAs, and most mRNAs have predicted targets for many miRNAs [22]. Up to now, many algorithms are publicly available for predicting miRNA-binding sites, including miRanda, TargetScan, PicTar, and miRBase [23, 24]. miRNAs regulate mRNA translation through recognition of binding sites of imperfect complementarity, in which pairing of miRNA nucleotides 2 to 8, or the seed region, is crucial. The seed region of nucleotides 2 to 8 at the 5′ end of the miRNA has perfect complementarity, and this sequence defines families of miRNAs. The miRNA sequence 3′ to the seed sequence has variable degrees of complementarity with the mRNA. miRNAs have been aptly referred to as “rheostats” because their regulatory impact is generally to fine-tune but not abolish protein expression [25].

## 3. MiR-223 Biogenesis and Function

The gene encoding miR-223 is located within the q12 locus of the X chromosome [26, 27], and its expression is regulated by several transcription factors, including transcription factor PU.1, CCAAT-enhancer-binding proteins- (C/EBP-) *α* and *β*, and nuclear factor I-A (NFI-A) [28–31]. Within the body, miR-223 appears to be most highly expressed in the bone marrow [28, 32], where its expression was shown to be restricted to the myeloid compartment [28]. The fact that the sequence of miR-223 has been remarkably conserved during evolution suggests that this miRNA has an important role in physiological processes. To date, miR-223's involvement has been demonstrated in many types of cancer, inflammatory disease, autoimmune disease, and other pathological processes [3].

The most important role of miR-223 was discovered in the field of hematology, since it was shown to modulate the differentiation of hematopoietic lineages [27]. This function takes place in the hematopoietic bone marrow and affects hematopoietic stem cells and myeloid, erythroid, and lymphoid cells at different stages during their development [27]. In bone marrow, miR-223 expression is mainly confined to myeloid cells and is induced during the lineage differentiation of myeloid progenitor cells. These myeloid cells will give rise to monocyte/macrophage and granulocyte cells. The level of miR-223 is reduced when granulocyte-monocyte progenitors start to differentiate into monocytes [31], whereas its level is increased when granulocyte-monocyte progenitors enter the granulocyte differentiation phase [27, 31], indicating a lineage-specific pattern. By contrast, using miR-223^−/−^ mice, the same group showed that miR-223 is not strictly essential for granulocyte differentiation but was required for normal maturation of granulocytes and regulation of the granulocyte compartment size [27]. In addition, miR-223 was shown to play a role in monocyte/macrophage differentiation, by targeting I*κ*B kinase subunit alpha (IKK-*α*), a component of nuclear factor-kappa B (NF-*κ*B) pathway. During macrophage differentiation, a fall in miR-223 expression induces an increase of IKK-*α* expression which induces the expression of p52 followed by the repression of NF-*κ*B pathways [33, 34]. Moreover, miR-223-rich microvesicles were suggested to induce the differentiation of recipient monocytes, activate hematopoietic cell production in the bone marrow, and then induce the release of more microvesicles [35]. Recently, miR-223 was demonstrated to regulate human embryonic stem cell (hESC) differentiation by targeting the IGF-1R/Akt signaling pathway [36]. The inhibition of miR-223 expression maintained hESCs in the undifferentiated state, while addition of exogenous miR-223 induced their differentiation. These effects were dependent on the IGF-1R/Akt pathway, due to the fact that IGF-1R mRNA was demonstrated to be a target of miR-223 [36].

The second field that miR-223 is actively involved in is angiogenesis and vascular remodeling, which may have therapeutic potential for certain cancer and cardiovascular disease. miR-223 is typically associated with myeloid cells, but recent investigation revealed that miR-223 is expressed in native endothelial cells and is rapidly downregulated after isolation and culture of the endothelial cells [37]. Additionally, miR-223 could attenuate endothelial cell proliferation by targeting the expression of *β*1 integrin and reducing the vascular endothelial cell growth factor- (VEGF-) induced and basic fibroblast growth factor- (bFGF-) induced phosphorylation of their receptors, as well as downstream Akt phosphorylation [37]. Accordingly, downregulation or deletion of miR-223 markedly increased *β*1 integrin expression, as well as angiogenesis and vascular repair. Therefore, it seems that miR-223 may be required for the maintenance of endothelial cell quiescence. It is interesting to determine whether the endothelial dysfunction associated with cardiovascular disease is also associated with altered endothelial miR-223 levels. Another intriguing, yet unproved, regulatory pathway of miR-223 in angiogenesis might be derived from recent evidence that hypoxia could downregulate the transcription factor CCAAT/enhancer binding protein-*α* (C/EBP-*α*) [38], which is also an upstream regulator for miR-223 expression [39]. A variety of cellular stresses, including replication stress and oxidative stress, play an important role in angiogenesis and vascular remodeling, which lead to the activation of DNA damage signaling [40]. Mounting evidence suggests that miRNA expression is regulated in response to DNA damage, and vice versa [41]. A recent work reported that an increased sensitivity to chemotherapy was observed in cancer cells with enforced miR-223 expression and reduced poly(ADP-ribose) polymerase-1 (PARP-1) [42], an enzyme which catalyzes the NAD^+^-dependent polymerization of long chains of poly-ADP ribose (PAR) onto itself in response to DNA damage, leading to DNA repair. Thus, miR-223 may serve as an endogenous PARP-1 inhibitor for cancer treatment. As DNA damage/repair response has been implicated in the pathogenesis of vascular remodeling [43, 44], future work is warranted to determine whether miR-223 may also participate in this process by targeting PARP-1.

The third field in which miR-223 was intensively investigated is osteoclast formation and bone remodeling [45]. Indeed, this miRNA is expressed in osteoclast precursors (RAW 264.7 cells); both under- and overexpression of miR-223 diminish the osteoclast-like cell formation induced by RANKL [29]. This indicates that the expression of miR-223 must be fine-tuned for normal osteoclastogenesis [29, 30, 45]. The third important role of miR-223 was in regulation of carcinogenesis [13, 46]. For example, miR-223 is poorly expressed in acute myeloid leukemia (AML), as well as in gastric [47] and ovarian cancer [48]. Depending on the context, miR-223 acts as either an oncogene or a tumor suppressor gene [49].

In addition to its role in hematopoietic differentiation, osteoclastogenesis, and embryonic stem cells differentiation, as well as in carcinogenesis, miR-223 also has an instrumental role in inflammatory diseases, such as adipocyte inflammation associated with morbid obesity, insulin resistance, rheumatoid arthritis, vascular damage, and atherosclerosis [3, 32, 49–52]. In brief, many studies show a clear trend towards a regulatory role of miR-223 in various clinical disorders. Further work will be warranted to determine the molecular mechanisms of miR-223 implicated in the physiopathology of these disorders.

## 4. Intraplatelet miR-223 and Platelet Reactivity

Platelet adhesion and aggregation play a pivotal role in the maintenance of hemostasis, as well as in thrombosis and vessel occlusion, which underlies the pathogenesis of stroke and acute coronary syndrome (ACS) [53]. Dual antiplatelet therapy with clopidogrel (a P2Y12 receptor antagonist) and aspirin is currently the cornerstone of pharmacological prevention of ischemic events in patients with atherosclerosis [54]. But epidemiological and clinical studies have identified a fraction of patients with increased risk of ischemic cardiovascular events despite receiving a standard regimen of antiplatelet therapy, which is termed “high on-treatment platelet reactivity” (HTPR) [55, 56]. Recent studies showed that HTPR is a strong predictor of myocardial infarction (MI) and cardiovascular death in patients after percutaneous coronary intervention (PCI) [57, 58]. Although heritability strongly influences the interindividual variation in platelet reactivity [59], the precious molecular mechanisms of this phenomenon remain to be poorly understood.

Platelets are devoid of a nucleus and genomic DNA, but they were shown to contain subcellular organelles, such as rough endoplasmic reticulum and ribosomes [60], as well as a small amount of poly(A) + RNA from their megakaryocyte progenitor cells [61], sufficient to support* de novo* protein synthesis. In fact, between 15% and 32% of protein coding genes are represented in the form of mRNAs in platelets [62–64]. In addition, the functionality of these platelet transcripts was supported by a strong correlation between transcript abundance and protein expression [63, 64]. These evidences prompted basic researchers to speculate whether circulating platelets harbor a gene-regulatory pathway based on miRNAs. This hypothesis was first confirmed by a landmark study by Landry and coworkers in 2009, which demonstrated that human platelets harbor an abundant and diverse array of miRNAs, and the three most abundant miRNAs are miR-223, let-7c, and miR-19a [65]. Further analyses revealed that platelets contain the Dicer and Ago2 complexes, which function in the processing of exogenous miRNA precursors and in the control of specific reporter transcripts, respectively. Accordingly, the authors could not detect the nuclear microprocessor components Drosha and DGCR8 in platelets, consistent with the anucleate nature of human platelets. They also found that miRNA-associated Ago2 proteins, in a complex reminiscent of recombinant RISC, may form the endogenous miRNA effector complex in platelets. Moreover, the presence of P2Y12 mRNA in Ago2 immunoprecipitates was detected, suggesting a potential regulation of P2Y12 expression by miRNAs in human platelets. Serving as a receptor for ADP, P2Y12 is a seven-transmembrane domain receptor coupled to Gi2 protein that mediates a number of biological processes, such as platelet aggregation, granule secretion, and thrombus growth and stability [66]. The experimental validation of the predicted binding site for miR-223 in its natural 3′UTR context in P2Y12 mRNA supports the concept that P2Y12 expression could be regulated by miR-223 in human platelets [65].

Another study by Nagalla and coworkers focused on the potential role of platelet miRNAs as both a biomarker of platelet reactivity and a regulator of platelet mRNA variation [67]. Using a genome-wide profiling strategy in 19 healthy donors, they demonstrated the expression of 284 miRNAs in platelets, among which miR-223 is present in greatest abundance. They then showed that miRNA profiles are associated with and may predict the response of platelet aggregation to epinephrine. In addition, the authors used computational approach to theoretically generate a high-priority list of miRNA-mRNA pair candidates, among which the functionality of three pairs (miR-200b:*PRKAR2B* (encoding the regulatory chain of cAMP-dependent protein kinase type II); miR-495:*KLHL5* (encoding a Kelch-like protein that binds to actin); and miR-107:*CLOCK* (encoding a major regulator of the cell circadian rhythm)) was further verified in cell lines [67]. Interestingly, a prior study by the same group showed that platelet vesicle-associated membrane protein 8 (VAMP8) expression (a protein involved in platelet granule secretion) is associated with differential platelet aggregation response to epinephrine in healthy donors, which is regulated by miR-96 [68]. These lines of evidence support a regulatory role of platelet miRNAs in protein expression that associated with variation in platelet reactivity.

Enlightened by the above studies, we hypothesized that the expression of miR-223 and miR-96, which are associated with platelet aggregation and secretion properties in healthy subjects, may influence the responsiveness to clopidogrel in patients with coronary heart disease (CHD). We thus enrolled 33 nondiabetic CHD patients with non-ST elevation acute coronary syndrome (NSTE-ACS) confirmed by diagnostic coronary angiography (CAG) [69]. All patients received a loading dose of 300 mg aspirin plus 300 mg clopidogrel for at least 24 h before CAG or 100 mg aspirin plus 75 mg clopidogrel for at least 5 days prior to CAG. Platelet responsiveness after antiplatelet therapy was determined by two methods (platelet reactivity index (PRI), measured by vasodilator-stimulated phosphoprotein (VASP) phosphorylation flow cytometry and ADP-induced platelet aggregation (PAG), measured by light transmission aggregometry). All patients were dichotomized according to the medians of their PRI and PAG values. We determined the expression of miR-223 and miR-96 in purified platelets from each patient and found that only miR-223 expression was statistically downregulated in PRI-determined low responders. In addition, neither miR-223 nor miR-96 was found to be significantly different between PAG-determined normal and low responders. Accordingly, miR-223 expression, but not miR-96, was statistically correlated with PRI. Using a binary logistic regression model, we showed that decreased miR-223 expression was the only variable that was associated with PRI-determined low responders, even after being adjusted for known risk factors for HTPR (*CYP2C19* ^*^ *2* genetic polymorphisms [59, 70], calcium channel blockers [71], proton-pump inhibitors [72], age, obesity, and smoking [73–75]). To our knowledge, this work provides the first evidence that platelet miR-223 may serve as a potential marker in evaluating the degree of P2Y12 receptor inhibition in CHD patients.

## 5. Circulating miR-223 and Platelet Reactivity

Despite the above evidence that platelet specific miRNAs are associated with varied response to antiplatelet therapy, the detection of intraplatelet miRNAs requires a large amount of blood (15–20 mL), as well as a white blood cell depletion protocol using magnetic beads, which may substantially limit its clinical application. Circulating miRNAs are stable in serum and plasma and were shown to have prognostic values for cardiovascular disease [76]. Currently, three pools of plasma circulating miRNAs have been identified, that is, microparticle- (MP-) associated, high density lipoprotein- (HDL-) associated, and exosome-associated miRNAs [77]. Among these three pools, it has been reported that a prominent amount of plasma miRNAs is associated to MPs [78].

The prognostic value of circulating miRNAs for major cardiovascular events (MACEs) was first confirmed by Zampetaki and coworkers [79]. In a cohort study consisting of 820 participants, this group found that baseline levels of three circulating miRNAs, that is, miR-126 (hazard ratio (HR) 2.69, 95% CI: 1.45–5.01), miR-223 (HR 0.47, 95% CI: 0.29–0.75), and miR-197 (HR 0.56, 95% CI: 0.32–0.96), were independently correlated with incident MI during a 10-year follow-up [79]. Notably, the authors showed that these miRNAs are highly expressed in platelets and platelet-derived MPs [79]. This finding is consistent with a recent study which showed that activated platelet-derived MP is the major source of plasma circulating miR-223, whereas HDL- and exosome-derived miR-223 is almost negligible compared with MPs [80]. Based on these findings, Willeit and colleagues extended their findings by profiling 377 miRNAs in human platelets, platelet MPs, and platelet rich and poor plasma and found that miR-223 is the most differentially expressed miRNAs [81]. Interestingly, the authors reported that the level of circulating miR-223 tended to be decreased after 10 mg prasugrel plus 75/300 mg aspirin in healthy volunteers (*n* = 3) and in patients (*n* = 15) with symptomatic carotid atherosclerosis after 300 mg clopidogrel plus 75 mg aspirin [81]. Thus far, the VASP index measured by flow cytometry is the most specific assay to evaluate P2Y12 receptor blockade and is not influenced by other antiplatelet agents or anticoagulants [82]. As the authors did not perform platelet VASP analysis, the relationship between platelet responsiveness after P2Y12 inhibition and the magnitude of miR-223 change remains unclear. In addition, as pointed out by the accompanying editorial [83], if miR-223 is suppressed by P2Y12 inhibition, its inverse relationship to incident MI seems obscure.

We recently extended our prior work by focusing on the prognostic value of circulating miR-223 for diminished response to clopidogrel in NSTE-ACS CHD patients [84]. In multivariate analysis, the level of circulating miR-223 independently predicted the differential response to clopidogrel after adjustment of known clinical factors associated with HTPR [84]. Admittedly, our study was not designed to investigate the influence of P2Y12 inhibition on circulating miR-223 level. However, the results from Zampetaki and colleagues [79, 81], as well as our independent work, indicate that circulating miR-223, presumably derived from platelets, may serve as a novel marker for platelet reactivity.

## 6. Controversy and Perspective

Based on multiple profiling studies providing evidence that miR-223 is one of the most abundant miRNAs in megakaryocytes and platelets [85–89], a recent work evaluated the platelet functions in miR-223 deficient mice [90]. Surprisingly, this study reported that, in miR-223-deficient mice, platelet number, volume, and lifespan, as well as platelet surface receptors, were expressed at the same level compared with those from wild type mice [90]. Moreover, loss of miR-223 did not affect the platelet properties for ADP-induced aggregation. In view of this evidence, the authors concluded that miR-223 plays a remarkably modest role in thrombopoiesis and that platelet function does not depend upon miR-223. This study seems contradictory to previous published human studies. However, an important issue should be noted. The central hypothesis that miR-223 is a biomarker for platelet reactivity is based on the evidence that there is a putative binding site for miR-223 in the 3′untranslated region (3′UTR) of the mRNA encoding P2Y12 [65], which is the molecular basis for miR-223 regulation of P2Y12 expression, whereas, as addressed in Leierseder and collaborators' recent work [90], the bioinformatics analysis revealed that, in contrast to the situation in humans, the mouse P2Y12 UTR does not contain a binding site for miR-223. Accordingly, they found that platelet P2Y12 mRNA expression was unchanged in miR-223 deficient mice compared with wide type mice. This finding pointed out a puzzling issue that the role of miR-233 may have substantial species difference and provided an explanation to the inconsistent results derived from human and mouse studies.

In addition to platelet's contribution to peripheral blood MPs, recent studies also demonstrated that mononuclear phagocytes, especially macrophages, are the second most abundant population contributing to MPs which lead to phenotype switch in target cells via miRNAs transfer, including miR-223 [35]. Moreover, miR-223 is highly expressed in cells of the granulocytic lineage and plays an important role in regulating granulopoiesis and neutrophil function [33]. Up to now, several miR-223 target genes that are closely associated with inflammation have been identified, such as NLRP3 [91], IKKa [34], Pknox1 [32], Pax6 [92], NFI-A [30], and insulin-like growth factor-1 receptor (IGF-1R) [93]. Therefore, it is possible that platelet-derived miR-223 may regulate other immune cells harboring miR-223 target genes via MP transfer and in turn regulate platelet function via platelet-leukocyte interaction (aggregate formation), a process now called immunothrombosis [94].

Appropriate regulation of platelet function, which is the basis to maintain the elegant balance between hemorrhage and thrombosis, is of paramount importance in patients on antiplatelet therapy. There is mounting evidence that platelet miRNAs mediated regulatory network serves as basic mechanisms for megakaryocyte/platelet gene expression. Given its abundance in platelet, miR-223 has begun to receive considerable attention. Recent studies provide an emerging role of miR-223 as both a regulator and biomarker for HTPR and MACEs. Bearing in mind that miR-223 mediated regulatory network may have species difference, future studies on miR-223 in different models of cardiovascular disease, as well as in clinical settings, will be warranted for developing new diagnostic and therapeutic strategies.
